# Reliability of the Actigraph GT3X+ Accelerometer in Adults under Free-Living Conditions

**DOI:** 10.1371/journal.pone.0134606

**Published:** 2015-08-14

**Authors:** Eivind Aadland, Einar Ylvisåker

**Affiliations:** Faculty of Teacher Education and Sport, Sogn og Fjordane University College, Sogndal, Norway; Universidad Pablo de Olavide, Centro Andaluz de Biología del Desarrollo-CSIC, SPAIN

## Abstract

**Background:**

Reliability of the Actigraph GT3X+ accelerometer has not been determined under normal wear time criteria in a large sample of subjects and accelerometer units. The aim of this study was to assess contralateral hip difference and inter-instrument reliability of the Actigraph GT3X+ monitor in adults under long-term free-living conditions.

**Methods:**

Eighty-seven adult subjects (28 men; mean (standard deviation) age 31.3 (12.2) years; body mass index 23.7 (3.1) kg/m^2^) concurrently wore two GT3X+ accelerometers (174 units in total) attached to contralateral hips for 21 days. Reliability was assessed using Bland-Altman plots, mixed model regression analyses and absolute measures of agreement for different lengths of data accumulation (single-day-, 7-day- and 21-day periods).

**Results:**

There were no significant differences between contralateral hips (effect size ≤0.042; p ≥.213). Inter-instrument reliability increased with increased length of data-accumulation. For a 7-day measurement period (n = 232 weeks), limits of agreement were ±68 cpm (vertical axis) and ±81.3 cpm (vector magnitude) for overall physical activity (PA) level, ±51 min for sedentary time, ±18.2 min for light PA, ±6.3 min for moderate PA, ±3.5 min for vigorous PA, and ±6.7 min for moderate-to-vigorous PA.

**Conclusions:**

The Actigraph GT3X+ accelerometer is a reliable tool for measuring PA in adults under free-living conditions using normal data-reduction criteria. Contralateral hip differences are very small. We suggest accelerometers be attached to the right hip and data to be accumulated over several days of measurement.

## Introduction

Despite some limitations, there are general agreement that accelerometry provide valid estimates of free-living physical activity (PA) and sedentary behavior (SED) in diverse study settings. Yet, as reliability is a premise for validity [1], determination of sources of measurement variability in accelerometer-measurements are important to judge their appropriateness to support various study findings. Importantly, whether PA is measured as a predictor (x-) variable or an outcome (y-) variable, noise will hamper researchers’ ability to make valid causal inferences in PA epidemiology [2] and possibly misinform the society regarding targets for public health management.

Intra- and inter-instrument reliability has been studied for a wide range of accelerometer makes and models using a range of study set-ups. Of these monitors, the Actigraph monitors (Actigraph, Pensacola, FL, USA, formerly known as Computer Science and Applications (CSA) and Manufacture Technology Incorporated (MTI) models) are the most used and studied, and have shown favorable reliability compared to other monitor brands [3–5]. The early models 7164 and GT1M have shown coefficients of variation (CV) of <5% for intra-instrument variation [5–7] and <8.9% for inter-instrument variation in mechanical- [5, 6, 8] and laboratory standardized PA set-ups [4]. However, only two small studies (n <15) have investigated inter-instrument variability for intensity-specific PA and SED under free-living conditions (24 hours), of which CVs ranged from 0.9 to 13.5% [9] and 3.0 to 10.5% [3] across outcome variables.

In 2009/2010 Actigraph released the tri-axial accelerometer GT3X/GT3X+ [10], which among other features allow for determination of the vector magnitude (VM) sum of movement over 3 axes. To the best of our knowledge, four studies have investigated the inter-instrument variability of the GT3X/GT3X+ accelerometer [11–14], of which all concluded that the monitor, in general, is reliable. Santo-Lozano et al found CVs from 2.2 to 201.8% (y-axis) (ICC 0.98) for 10 GT3X units in a mechanical set-up [11], and CVs from 1.4 to 20.4% (y-axis) (ICC 0.93–1.00) and 1.1 to 22.3% (VM) (ICC 0.95–1.00) for 8 GT3X units attached to the contralateral hips of a single person applying standardized PAs [13]. Ozemek et al [12] found a mean ICC of 0.94 (y-axis) over 20 activities of daily living in 40 subjects using 2 GT3X+ units attached to the same hip. Finally, Jarrett [14] found CVs from 1.5 to 18.2% (ICCs > 0.97) for 24 h free-living PA in 19 subjects using 20 GT3X+ units attached to contralateral hips. Despite the apparently excellent reliability for total PA level (CV 2.8%; ICC 0.99) and moderate-to-vigorous PA (MVPA) (CV 2.9%; ICC 0.99), 95% limits of agreement (LoA) indicated that variation was substantial, amounting to -82 to 109 counts per minute (cpm) and -6.1 to 18.7 min/day for total PA level and MVPA, respectively.

The current studies that have investigated the inter-instrument reliability of the GT3X/GT3X+ monitor have several critical limitations. First, findings are based on relatively few subjects and/or monitors, which question the validity and generalizability of their findings. Secondly, free-living and intensity-specific PA have only been investigated in a single study [14] using a short (24 hour) measurement period, thus, the authors calls for studies applying a longer period of measurement. Thirdly, studies of the Actigraph models 7164, GT1M and GT3X+ have pointed toward different intensity-categories to introduce the most variability between instruments [3, 9, 14]. This might be explained by the small number of observations (n ≤19), leaving findings susceptible to random variation. Finally, the findings from the study by Jarrett et al [14], using a contralateral hip design, might be confounded by differences between the right and left hip (or dominant and non-dominant side), as significant differences between hips have been detected in some studies [9, 13].

The aim of the present study was to assess the contralateral hip difference and the inter-instrument reliability of the Actigraph GT3X+ monitor to determine overall PA level as well as intensity-specific PA and SED in adults under free-living conditions. We included a 21-day measurement period in 87 subjects and applied a total of 174 accelerometer units. We hypothesized that the inter-instrument reliability would improve along with an increased length of the measurement period, and that there would be no difference between contralateral hips.

## Material and Methods

### Subjects

Eighty-seven subjects were recruited by word of mouth mainly among students and staff at the Sogn og Fjordane University College, Norway for a long-term (21 days) objective measurement of PA level. Students and staff at the Sogn og Fjordane University College received oral and written information regarding the study. Accelerometers were thereafter made available for pick-up and later returned at Campus, using a “mail-box”. Thus, the data was collected anonymously. The study was reviewed by the Regional Committee for Medical and Health Research Ethics of Western Norway, who confirmed that their approval was not required.

### Procedures

Physical activity was measured using a total of 174 Actigraph GT3X+ accelerometers (firmware 2.2.1) (Pensacola, FL, USA). Two accelerometer units were attached to an elastic belt, and worn at contralateral hips over a 21-day period. Half-way through the measurement period (day 11), subjects switched the accelerometer units around (1 unit was worn on the right hip the first 10 days and thereafter at the left hip the last 10 days, and vice versa). Thus, each accelerometer unit was worn at both hips. This procedure allowed for an accurate analysis of differences between hips, as we avoid confusion of differences between hips and differences between accelerometer units. Subjects were instructed to wear the accelerometers at all times, except during water activities (swimming, showering) or while sleeping. The accelerometers were initialized at a sampling rate of 30 Hz. Files were analyzed at 10 second epochs using the Kinesoft v. 3.3.75 software [15]. A wear time of ≥480 minutes/day was used as the criterion for a valid day, and ≥3 and 9 days (i.e. a mean of ≥3 days per week) were used as the criteria for a valid 7-day and 21-day period of accumulated data, respectively. Consecutive periods of ≥60 minutes of zero counts (allowing for ≤2 minutes of non-zero counts) were defined as non-wear time and excluded from the analyses [16, 17]. Inter-instrument reliability was investigated for the following variables obtained from the vertical axis; wear time, overall PA (i.e., counts per minute: CPM), SED (<100 cpm), light PA (LPA) (100–2019 cpm), moderate PA (MPA) (2020–5998 cpm), vigorous PA (VPA) (≥5999 cpm) and MVPA (≥2020 cpm) [18], as well as the overall PA level based on the vector magnitude (VM CPM).

Subject characteristics (sex, age, body mass and height) were self-reported. Body mass index (BMI) was calculated as the body mass divided by the squared height (kg/m^2^).

### Statistical analyses

Subject characteristics were reported as means and standard deviations (SD).

Agreement was assessed by determining a) the mean difference (bias) between contralateral hips and b) the measurement error between the paired accelerometer instruments. The difference between the contralateral hips was analyzed using a linear mixed model regression analysis including a random intercept for subjects, including a dummy variable indicating a) the right versus the left hip and b) the dominant versus the non-dominant hip in separate analyses. Day 11 was excluded from these analyses, leaving a total of 2711 days for analysis across subjects and accelerometer units. Residuals were normally distributed in all models. Findings were reported as regression coefficients and 95% confidence intervals (CI), along with effect sizes (estimated difference/pooled SD).

The inter-instrument agreement (i.e., the difference between accelerometer units) was analyzed using three different time intervals for accumulating data: 1) Single days of measurement; 2) Three 7-day periods of measurement; 3) The total 21-day period. Thus, in the first and second analysis, up to 3 weeks and 21 days of measurement were included for each subject, respectively. Error was determined using Bland Altman plots, showing the difference between two accelerometer units as a function of the mean of the two units [19], and by absolute measures of agreement. Because the data were deemed to be homoscedastic (i.e., y not related to x in the Bland Altman plot), the standard error of the measurement (SEM) and 95% limits of agreement (LoA) were calculated according to Hopkins [20] (SEM = SD of the differences/√2; LoA = SD of the differences*1.96). In addition, the percentage typical error was calculated as SEM/mean values*100. The Pearson correlation coefficient (r) was also reported.

All analyses were performed using IBM SPSS v. 20 (IBM SPSS Statistics for Windows, Armonk, NY: IBM Corp., USA). A p-value < .05 indicated statistically significant findings.

## Results

All 87 subjects (28 (32%) men and 59 women; mean (SD) age 31.3 (12.2) years; body mass 70.4 (12.2) kg, height 172.1 (8.1) cm, body mass index 23.7 (3.1) kg/m^2^) was included in the analyses.

In total, 1397 (76%) days, 232 (89%) weeks and 80 (92%) 3-week periods were valid according to the criteria applied, and included in the analyses. Table 1 shows the sample characteristics regarding PA, based on weekly accumulated data (6, 8, 18, 25 and 43% of the sample had 3, 4, 5, 6 and 7 valid days of data, respectively).

**Table 1:** Sample characteristics for physical activity level and sedentary time.

	Mean (SD)
Wear time (min/day)	773 (90)
CPM (cpm)	473 (179)
VM CPM (cpm)	878 (245)
SED (min/day)	536 (66)
LPA (min/day)	172 (58)
MPA (min/day)	56 (24)
VPA (min/day)	8 (9) [5 (8)]*
MVPA (min/day)	64 (27)

Physical activity level and sedentary time of the sample based on weekly accumulated data. CPM = counts per minute; VM CPM = vector magnitude counts per minute; SED = sedentary time; LPA = light physical activity; MPA = moderate physical activity; VPA = vigorous physical activity; MVPA = moderate-to-vigorous physical activity;

*Median (IQR) are reported for VPA due to skewed distributions.

Evaluation of differences between contralateral hips (right (n = 1361 days)–left (n = 1350 days) showed very small (ESs <0.042) and no statistically significant differences (Table 2). Differences between the dominant and non-dominant hip were similar to the results for the right vs. left hip (ESs <0.015; p ≥.555).

**Table 2:** Differences between contralateral hips.

	Difference (95% CI)	*p*	ES
Wear time (min/day)	5.5 (-3.2 to 14.3)	.213	0.040
CPM (cpm)	-7.6 (-24.7 to 9.5)	.382	0.028
VM CPM (cpm)	5.8 (-17.0 to 28.6)	.619	0.016
SED (min/day)	4.7 (-2.8 to 12.3)	.216	0.042
LPA (min/day)	1.2 (-2.5 to 4.9)	.513	0.016
MPA (min/day)	-0.2 (-2.6 to 2.2)	.858	0.005
VPA (min/day)	-0.2 (-1.1 to 0.7)	.641	0.014
MVPA (min/day)	-0.4 (-3.1 to 2.2)	.745	0.009

The differences between contralateral hips (right—left hip) as analyzed with linear mixed model regression analyses based on single days of measurement (n = 1361 and 1350 days for the right and left placement, respectively). CI = Confidence Interval; ES = effect size (difference/pooled SD); CPM = counts per minute; VM CPM = vector magnitude counts per minute; SED = sedentary time; LPA = light physical activity; MPA = moderate physical activity; VPA = vigorous physical activity; MVPA = moderate-to-vigorous physical activity

**Fig 1:**
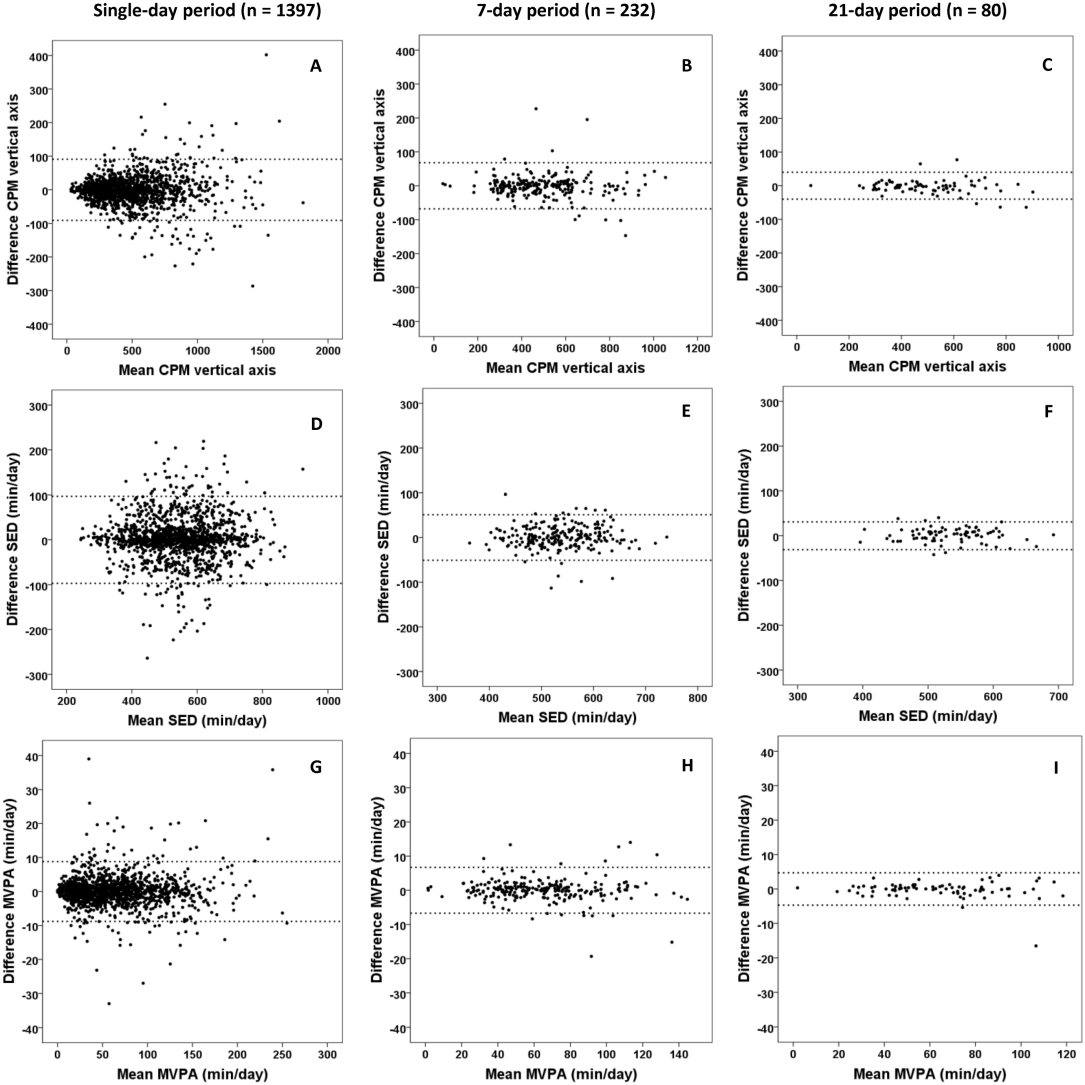
Bland Altman plots (the difference (right—left) between two accelerometer units on the y-axis versus the mean of the two units on the x-axis) for average counts per minute (CPM) from the vertical axis (a-c), sedentary time (SED) (d-f) and moderate-to-vigorous physical activity (MVPA) (g-i) for single days of measurement (n = 1397 person-days), 7-day periods of measurement (n = 232 person-weeks) and 21-day periods of measurement (n = 80). 95% limits of agreement are indicated as dotted lines.

The correlation between accelerometer units 1 and 2 were generally very high (r = 0.90–0.99, r = 0.93–0.99, and r = 0.96–1.00 for the single-day, 7-day and 21-day measurement periods, respectively). As shown in Table 3 and Fig 1, the measurement error decreased when PA was accumulated over a 7-day period (14–48% improvement) and a 21-day period (47–69% improvement) compared to single days of measurement, and when a 21-day period was compared to a 7-day period (25–52% improvement). For example, whereas units must be expected to differ by ±91 cpm for overall PA level over single days, this difference is reduced to ±68 and ±40 cpm when data are accumulated over a 7-day (i.e. 3 to 7 days) and 21-day (i.e. 9 to 21 days) period. For a 7-day measurement period typical errors were found to be in the range of 1.7 to 4.9% of the respective mean values, except for VPA which had a very small mean value leading to a magnified percentage error.

**Table 3:** Inter-instrument agreement for single days versus accumulated data.

	Length of data accumulation					
	Single-day (n = 1397)		7-day (n = 232)		21-day (n = 80)	
	SEM (%)	95% LoA	SEM (%)	95% LoA	SEM (%)	95% LoA
Wear time (min/day)	36.6 (4.7)	101.3	19.7 (2.6)	54.7	12.3 (1.6)	34.1
CPM (cpm)	32.7 (6.6)	90.6	24.5 (4.9)	68.0	14.4 (2.9)	39.8
VM CPM (cpm)	34.3 (3.9)	95.1	29.3 (3.3)	81.3	14.9 (1.7)	41.4
SED (min/day)	35.1 (6.5)	97.2	18.4 (3.4)	51.0	11.2 (2.1)	31.0
LPA (min/day)	10.1 (5.8)	28.0	6.6 (3.8)	18.2	3.2 (1.9)	8.8
MPA (min/day)	3.5 (6.3)	9.8	2.3 (4.0)	6.3	1.7 (3.0)	4.7
VPA (min/day)	1.6 (20.7)	4.5	1.3 (16.2)	3.5	0.8 (9.6)	2.2
MVPA (min/day)	3.2 (5.0)	8.8	2.4 (3.7)	6.7	1.7 (2.6)	4.7

The inter-instrument agreement for different measurement periods of accumulating the data (single-days of measurement, and data accumulated over 7-day-periods and a 21-day period). SEM (%) = standard error of the measurement (% of mean values); LoA = limits of agreement; CPM = counts per minute; VM CPM = vector magnitude counts per minute; SED = sedentary time; LPA = light physical activity; MPA = moderate physical activity; VPA = vigorous physical activity; MVPA = moderate-to-vigorous physical activity

## Discussion

The present study determined the inter-instrument reliability for the measurement of free-living PA and SED over 21 days using 174 Actigraph GT3X+ accelerometer units in 87 subjects. Thus, to the best of our knowledge, it is the first study that has investigated the inter-instrument reliability of many GT3X+ accelerometer units under normal conditions of measurement (i.e., free-living PA over several days) in a relatively large sample. For a 7-day measurement period, output from accelerometer units differed by (95% LoA) ±68 CPM, ±51 minutes of SED and ±6.7 minutes of MVPA. Inter-instrument reliability decreased for a shorter period of measurement, and improved when measurements were accumulated over a longer time-frame.

Contrary to our findings, significant differences in accelerometer output between contralateral hips have been detected in some previous studies [9, 13], but not all [14]. McClain [9] found a difference of 2 min of MVPA/day, however, the standardized difference were minimal (ES = 0.07) and quite similar to the differences detected in the current study (ES ≤0.042). Thus, in support of our hypothesis, there does not seem to be any meaningful difference in PA and SED as measured at the contralateral hips. Still, differences between hips might be found on an individual level. Thus, we suggest previous findings [9, 13] could have been subject to a type 1 error due to a random draw of relatively few subjects and monitors, thus being flawed by methodological shortcomings. The comparison made in the present study were not influenced by possible differences between accelerometer units, as the switch of side half-way through the measurement period (unit 1 worn at the right hip the first 10 days, switched on day 11 and worn on the left hip the last 10 days and vice versa) allowed for a within-unit controlled analysis of differences between the right and the left hip. This design in combination with a relatively large sample and 21 days of monitoring provide a high power to detect any difference. Thus, the findings supported our study hypothesis, meaning that a recommendation regarding which side of the body the accelerometer should be attached, is not appropriate based on our findings. For the same reason, when data are accumulated over several days, a day-by-day inconsistent attachment (right vs. left) is also of little importance. However, possible individual differences would call for a standardized attachment for repeated measurements (e.g., pre- and post-measurement). Thus, consistent with Ward et al [21], we suggest one side (right) be used for consistency.

The clear pattern of improved inter-instrument reliability along with increased length of data accumulation found in the present study supported the hypothesis put forward. To the best of our knowledge, we are the first to report findings on inter-instrument reliability having measurements over periods longer than 24 hours. This is an important finding, as inclusion of ≥3–4 days of valid wear time is recommended to obtain valid results from accelerometer measurements [16]. Interestingly, the finding of LoA of ±90.6 CPM for single days of measurement, is very similar to the finding by Jarrett et al [14] (~ ±95 cpm) using a 24 hour measurement period. The results of that study and the current study were also comparable for derived variables. However, our findings showed a clear reduction in variability when data were accumulated over a longer period, resulting in reductions in LoA of 14 to 48% to a 7-day period and 47 to 69% to a 21-day period. Although we generally do not find 21-days of monitoring feasible, the inclusion of such a long-term measurement serve the purpose of clearly illustrating the improved reliability obtained as a result of accumulating data over a longer time-period.

The improved reliability with increased accumulation of data is in accordance with probability theory and the law of large numbers, that is, the average result will tend to be closer to the expected value when the number of observations increases. Specifically, as there is a certain amount of intra-unit variation over time [5–7], the single-day measures are probably influenced by intra-unit-, as well as inter-unit variation. We believe accumulation of data over several days, thus cancelling out random intra-unit variation, are the key explanation for the finding of improved reliability for longer-term measurements. Therefore, we recommend accelerometer measurements be performed over several days, as recommended by others [16], to keep both intra-subject and inter- (and intra-) instrument variability to a minimum.

Previous studies assessing free-living intensity-specific PA and SED have detected variation in inter-instrument reliability over different intensity-categories. The greatest variation has been shown for SED (CV 10.5%) [3], MPA (CV 13.5%) [9], and VPA (CV 12.3%) and very VPA (CV 18.2%) [14]. Similar to Jarrett et al [14], we found the greatest percentage variation (SEM % of the mean values) for VPA. As shown in Fig 1, the distribution of the data was mainly homoscedastic (i.e., differences did not depend on the mean value), possibly except for axis 1 CPM (Fig 1A) where a heteroscedastic distribution could be argued. Thus, appropriate measures of reliability are SEM and LoA, as opposed to situations having heteroscedastic distributions, where CVs are the appropriate measure [22]. Thus, a great percentage variation for VPA could be explained by the very low mean value, despite absolute variation for VPA being less than for MPA in the current study (SEM 1.3 vs. 2.3 min/day for a 7-day measurement period, respectively), as well as the study by Jarrett et al [14]. Besides this statistical artifact, we found no evidence for a differential classification error, as hypothesized by McClain et al [9]. The lower percentage variation for VPA found by McClain, compared to the current study, could be explained by their sample consisting of runners, exhibiting a mean VPA of 47 min/day. Moreover, according to the authors, their sample being runners may have introduced the low reliability found for MPA, as subjects spent about twice the amount of time in VPA, compared to MPA, thus introducing a differential classification error between these categories [9]. Thus, such findings would not be expected to be seen in public health studies, where levels of VPA are low [23, 24]. The present study might support Jarrett et al’s hypothesis, as we found no evidence for a differential classification problem for MPA when MPA were clearly higher than VPA. Contrary to Vanhelst et al [3], we did not find evidence for lower reliability for SED compared to other variables. Yet, it should be noted that variation in SED could arise from variation in wear time and possibly number of valid days between accelerometers, as shown in the present study. Otherwise, we have no other explanation for these apparent discrepancies between studies than hypothesizing that the findings might arrive from random sampling of observations due to small sample sizes (n < 20) in previous studies [3, 9, 14].

To arrive at a decision whether a measure is reliable and fulfills analytical goals for a given study is a difficult case. Although some seems to choose some arbitrary cut point for acceptable reliability (e.g., CV <10%), a measure cannot be “significantly reliable”, thus leaving this decision to a matter of opinion and judgment [19, 22]. As noise in predictor (x-) variables will lead to attenuation of regression coefficients by biasing coefficients towards the null (regression dilution bias), and noise in outcome (y-) variables will increase standard errors and increase the likelihood of performing type II errors [2], unreliable measures will weaken researchers ability to make valid conclusions in PA epidemiology. Still, in line with previous studies, we argue that the Actigraph GT3X+ accelerometer is a reliable instrument for measuring overall PA level and intensity-specific PA. Moreover, previous studies have found inter-instrument variability to account for a very modest part of total variance in PA levels between subjects under laboratory (<6%) [4, 25] and field (<4.2%) [8] conditions. Nevertheless, inter-instrument variability should be kept in mind as one of several sources of variation in accelerometer measurements of PA.

### Strengths and limitations

The present study has several strengths. First, we included a large sample of subjects and monitors compared to previous studies. Thus, results are likely to be generalizable to a normal adult population and to the general pool of Actigraph GT3X+ accelerometers. Second, whereas previous studies investigating inter-instrument reliability under free-living conditions were limited to 24 hour monitoring periods, subjects in the present study wore the accelerometers for a long period (21 days). As our findings clearly showed that the inter-instrument reliability increased along with increased time of monitoring, the study provides researchers important insight into the relationship between monitoring length and reliability. Third, the comparison of contralateral hips was performed using a within-accelerometer controlled design. Thus, we were able to determine differences between hips per se.

Possible limitations to the present study include the use of multiple observations from the same subjects in analyses of single-day- and 7-day periods of measurement, and the inclusion of an adult sample. We used mixed-model analyses to account for clustering (adjustment of standard errors) when determining effect-estimates for differences between contralateral hips. Such a procedure is not straight-forward for reliability-analyses of random errors, where individual observations are modeled (as opposed to group means). Evaluation of the dependency of observations revealed low levels of clustering (intra-class correlation (ICC) <3.5% for single-day- and ICC <5.8% for 7-day periods of measurement). Inclusion of a random effect for subjects was not indicated for the 7-day measurements, as evaluated using the likelihood ratio test [26]. For single-days of measurement, a somewhat improved model fit was found when a random effect for subjects was included. Still, we believe clustering provide a negligible challenge to the interpretation of the present data. Due to the inclusion of an adult sample, future research might investigate inter-instrument reliability in other samples, although it may be argued that intra-subject comparisons of monitors may not differ considerably across population characteristics.

## Conclusion

The Actigraph GT3X+ accelerometer is a reliable tool for measuring overall PA level and intensity-specific PA and SED in adults under free-living conditions using normal data-reduction criteria. Inter-instrument reliability increased when accelerometer data were accumulated over a longer time-period. Furthermore, contralateral hip differences were minimal. Although inter-instrument differences are minor, the differences between units should be recognized, because noise in any measurement will attenuate “real” relationships between PA and health and increase the likelihood of performing type II errors. Along with current recommendations, we suggest accelerometers be attached to the right hip for consistency and data to be accumulated over several days of measurement.

## Supporting Information

S1 Dataset Supplementary data file including all material underlying the present study. (XLSX)
